# Supplementary material for: V-Dock: Fast Generation of Novel Drug-like Molecules Using Machine-Learning-Based Docking Score and Molecular Optimization
Source: Int J Mol Sci. 2021 Oct 27;22(21):11635. doi: 10.3390/ijms222111635 (PMC8584000; doi:10.3390/ijms222111635)
Supplement: Supplementary file 1 [file ijms-22-11635-s001.zip › ijms-1403625-supplementary.pdf]

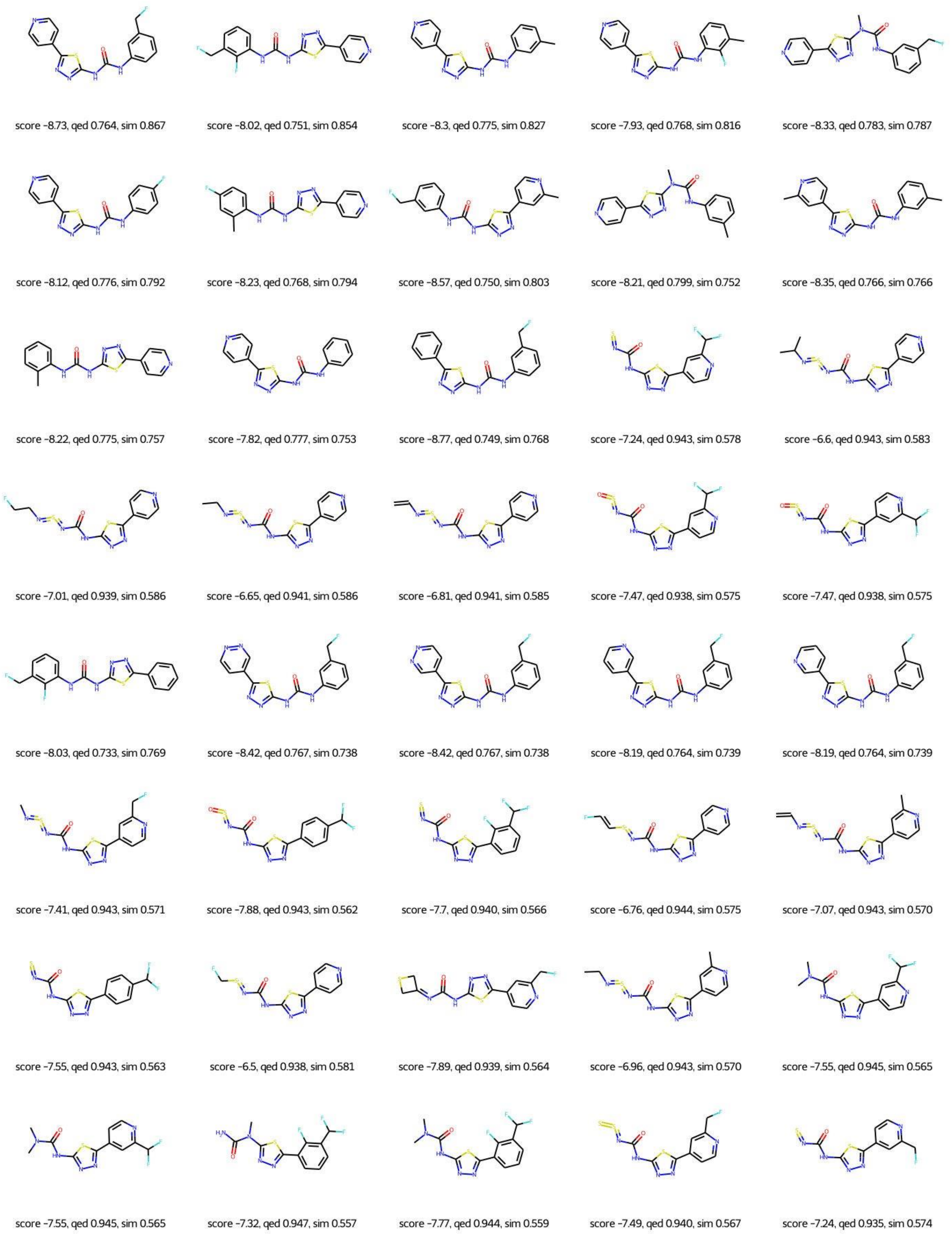

Figure S1. Top 40 Molecules Generated by MolFinder (ωD=0.01).

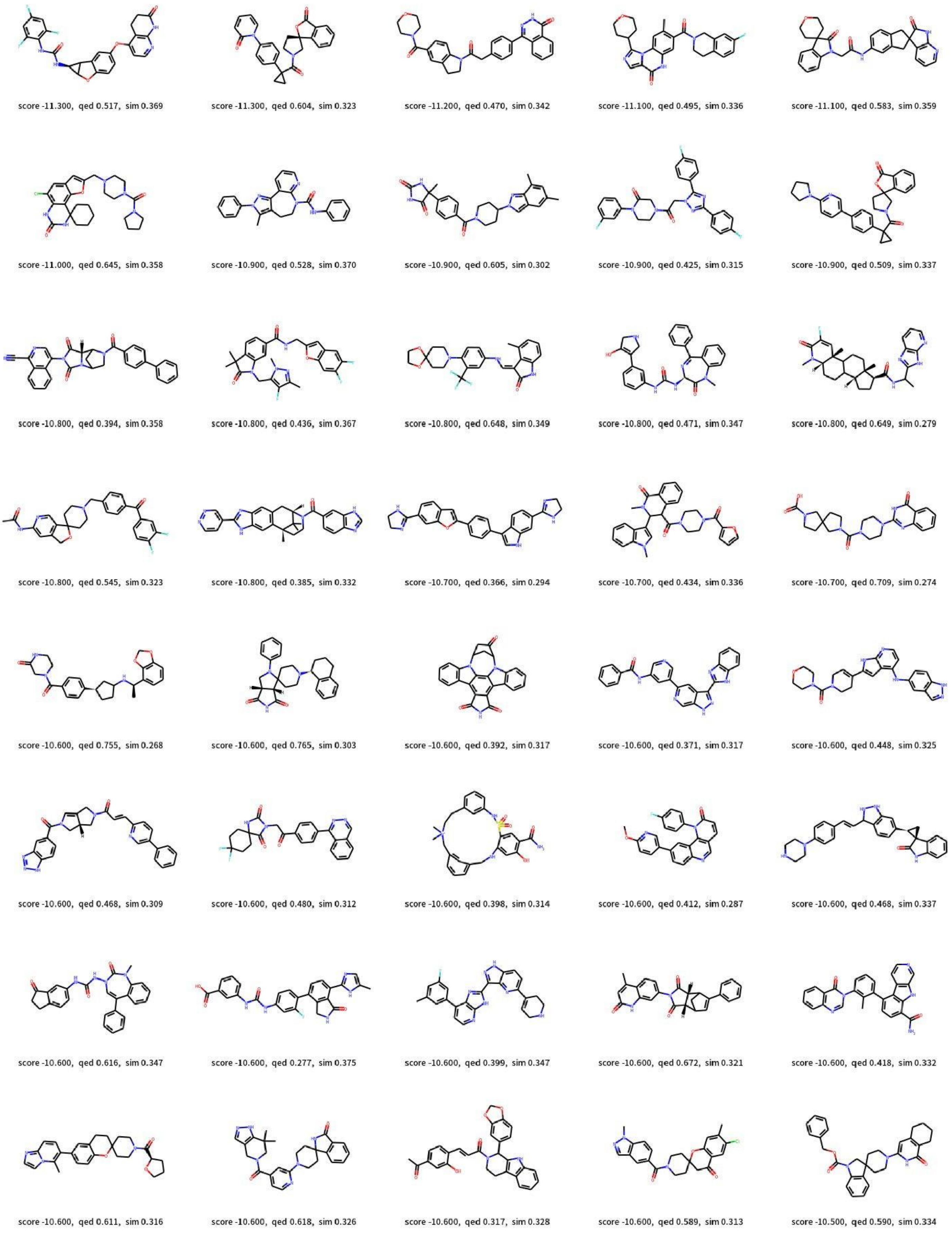

Figure S2. Top 40 molecules with the lowest docking energy and passing Lipinski's rule.

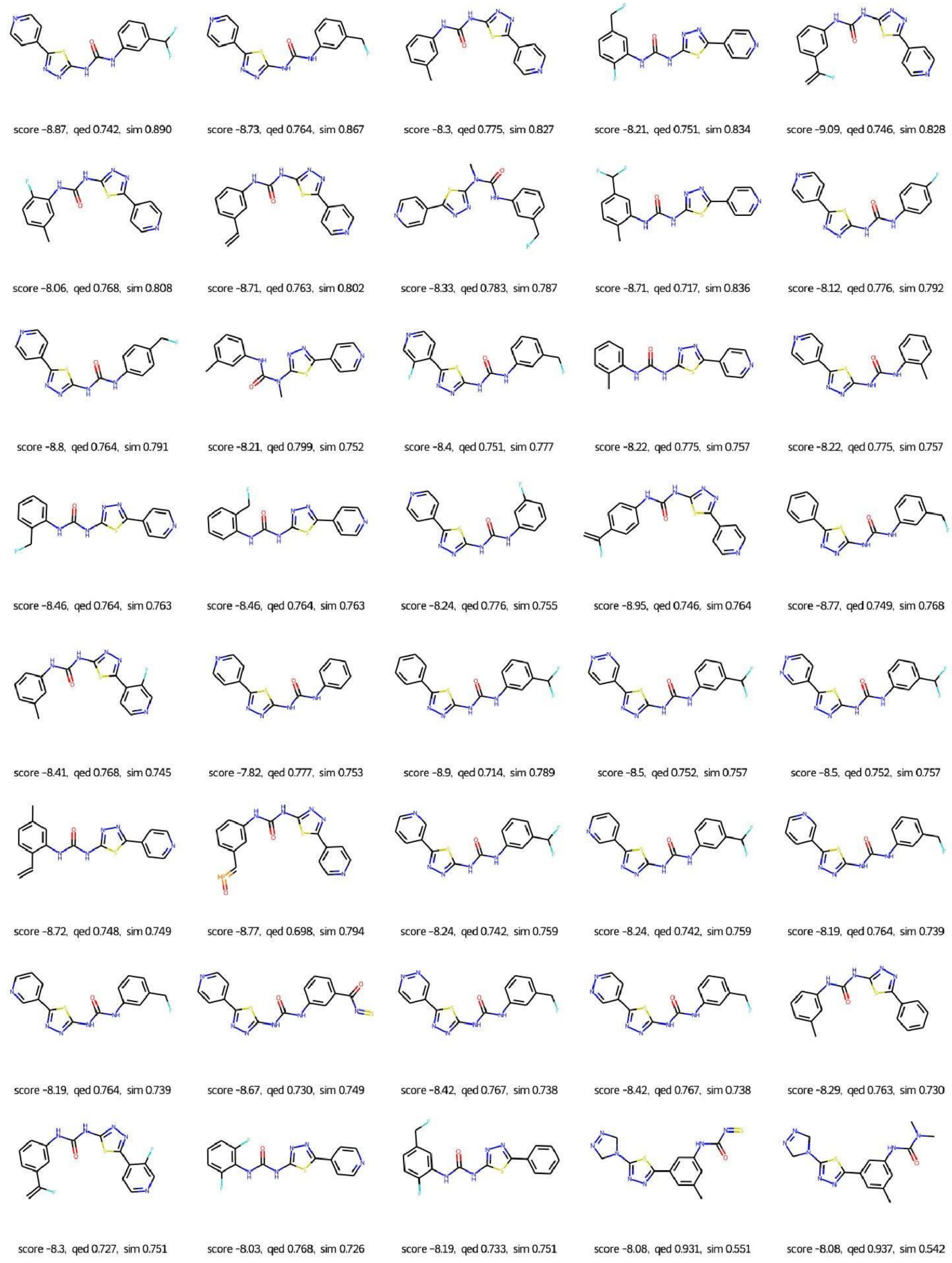

Figure S3. Top 40 Molecules Generated by MolFinder (ωD=0.03).

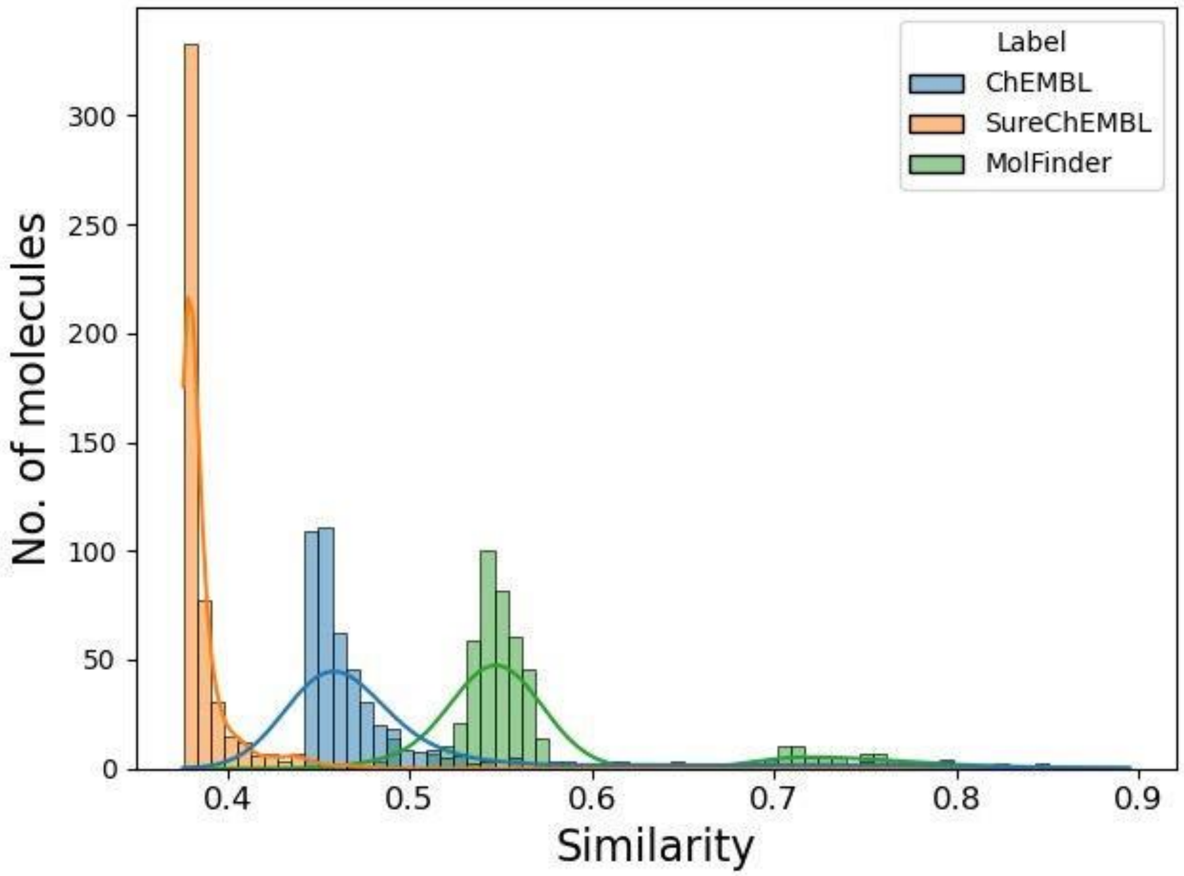

Figure S4. Comparison of similarity between generated molecules and ChEMBL and SureChEMBL data sets using MolFinder.From the ChEMBL and the SureChEMBL dataset, 500 molecules with the highest similarity to the reference molecule were selected and compared with the generated molecules using MolFinder. Molecules generated using MolFinder have values between 0.5 and 0.6, and they are more similar to the reference molecule than other data sets.

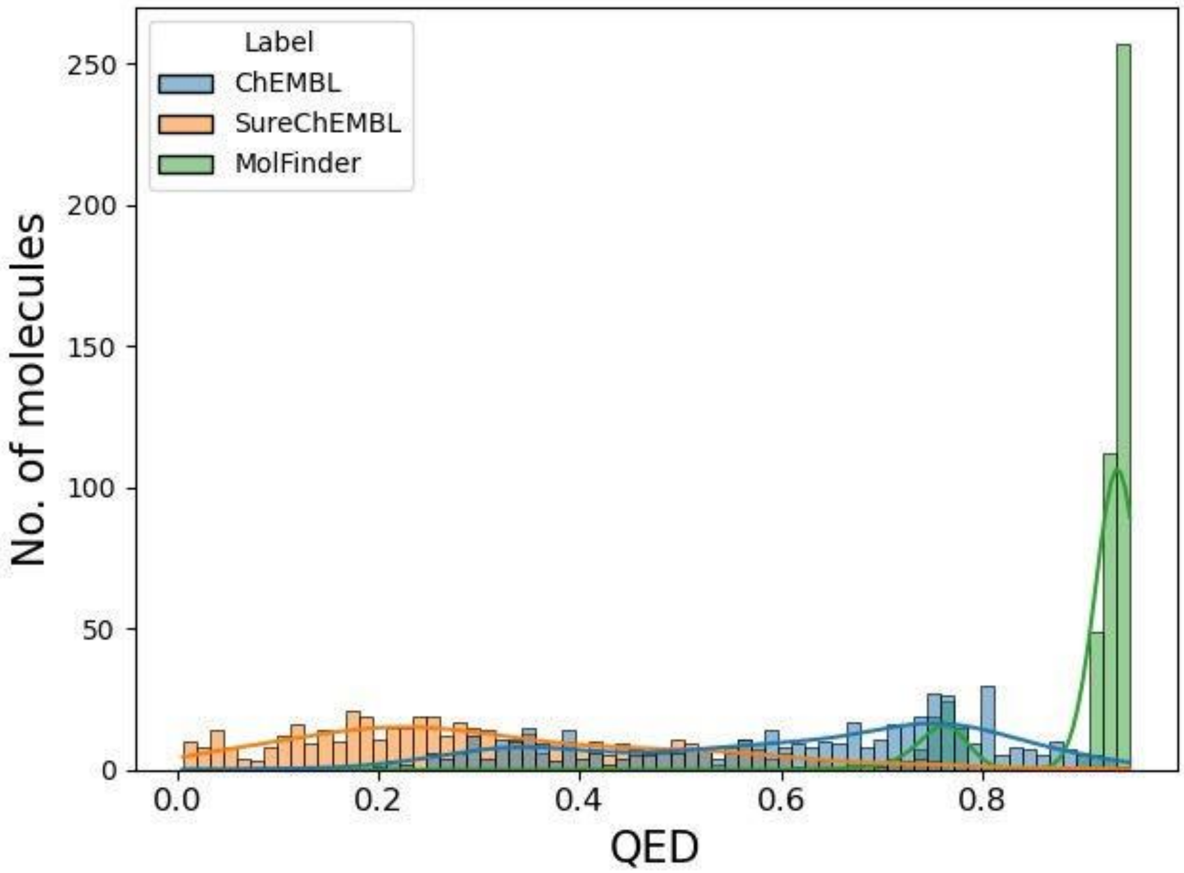

Figure S5. Comparison of QED between generated molecules and ChEMBL and Sure- ChEMBL data sets using MolFinderFrom the ChEMBL and the SureChEMBL dataset, 500 molecules with the highest similarity to the reference molecule were selected and compared with the generated molecules using MolFinder. Molecules generated using MolFinder have QED values between 0.65 and 0.95, and other data sets have a wide distribution of QED values.

Average of QED values

- - $S(m;m_{ref})$ - $Q(m)$ + 0.005 $D(m)$: 0.92
- - $S(m;m_{ref})$ - $Q(m)$ + 0.01 $D(m)$: 0.91
- - $S(m;m_{ref})$ - 0.50 $Q(m)$ + 0.005 $D(m)$: 0.84
- - $S(m;m_{ref})$ - 0.50 $Q(m)$ + 0.01 $D(m)$: 0.81

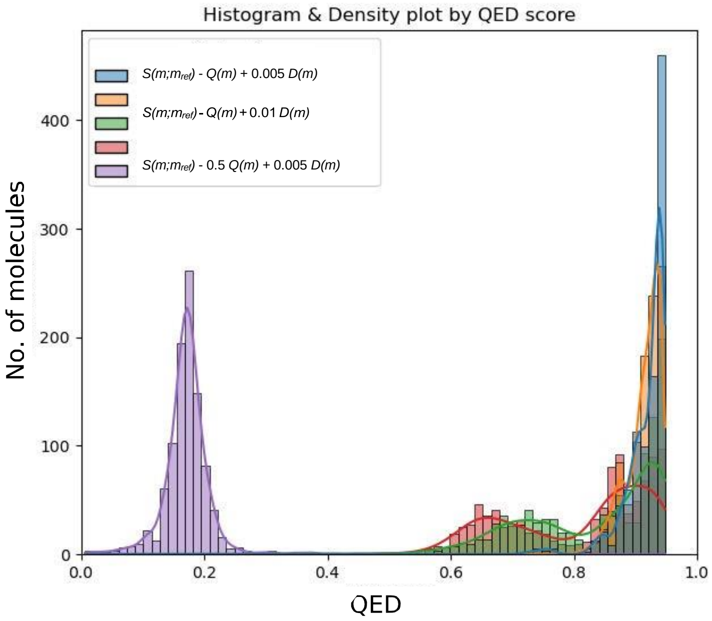

Figure S6. Generated molecules with the known molecules in SureChEMBL while varying the weight coefficient of the QED term (ω_Q = 1.0 and ω_Q = 0.50).

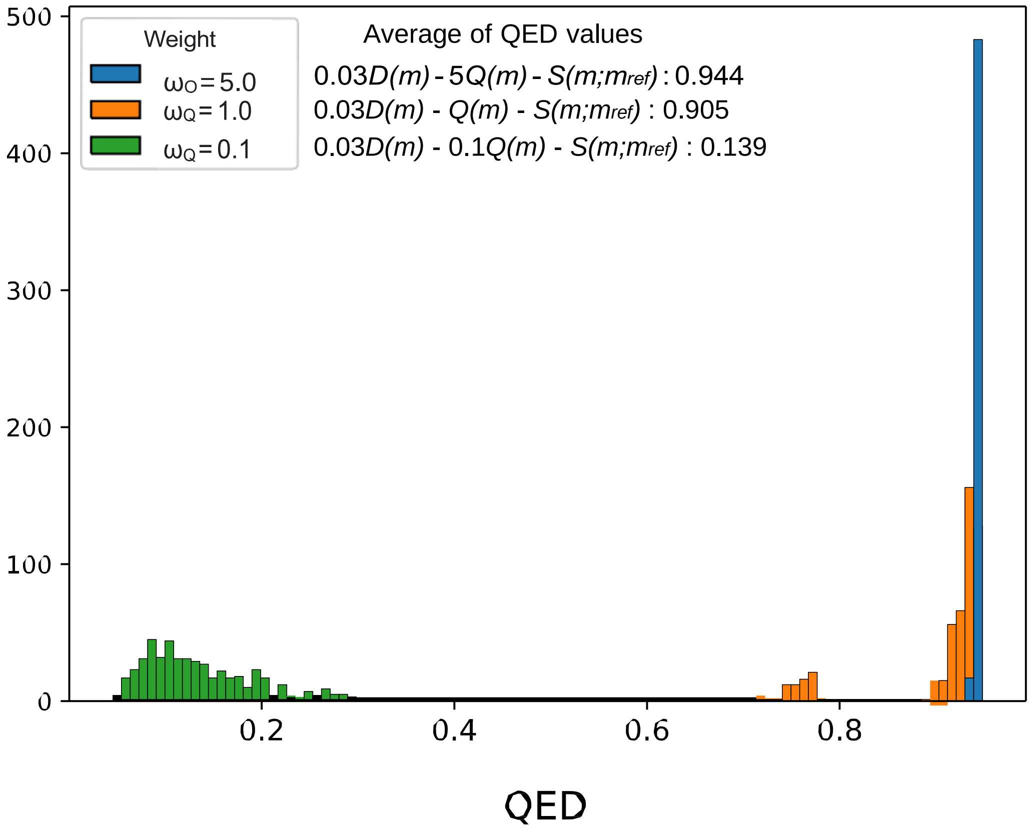

Figure S7. Generated molecules with the known molecules in SureChEMBL while varying the weight coefficient of the QED term (ωQ = 5.0, ωQ = 1.0, ωQ = 0.1).

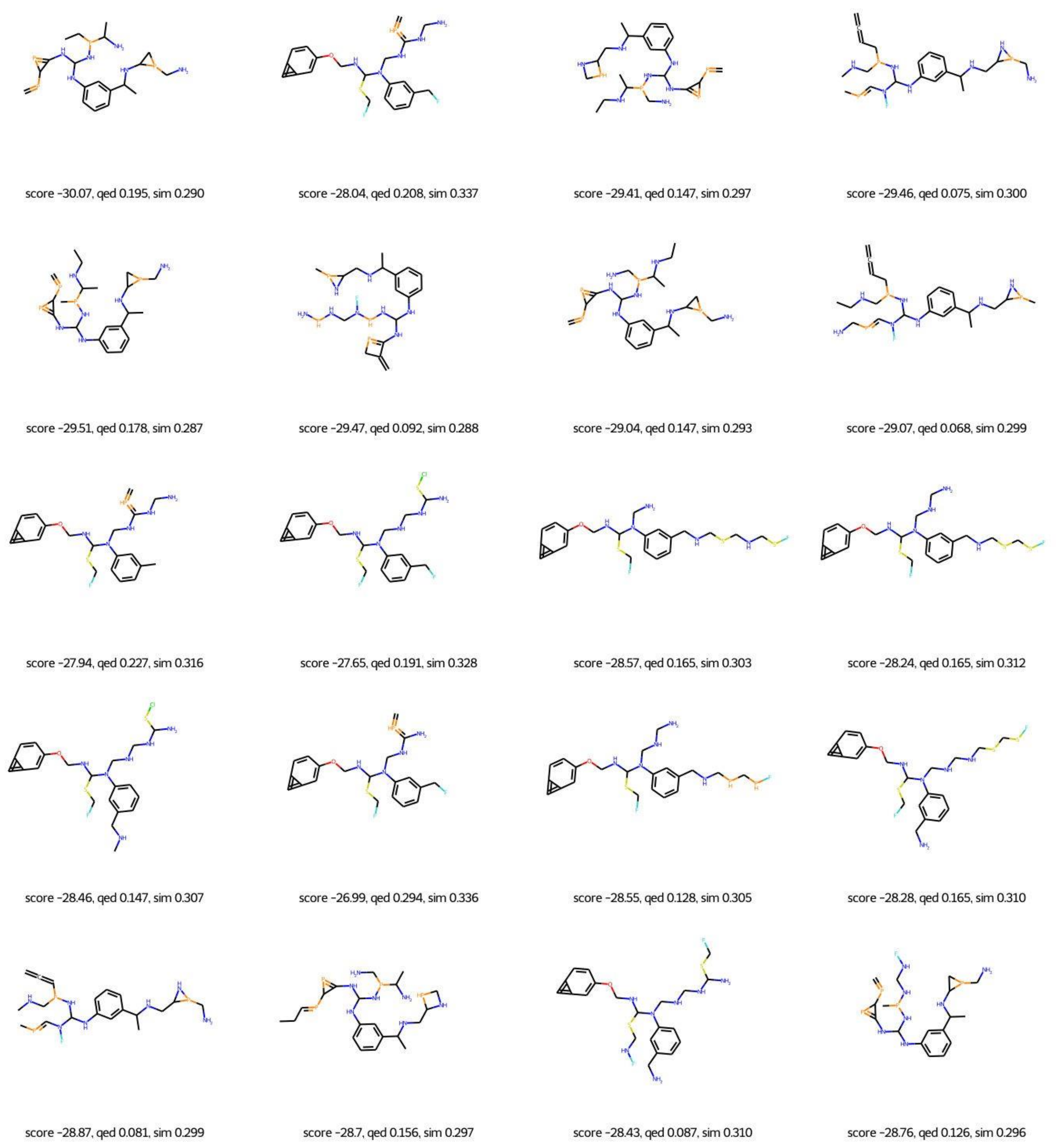

Figure S8. Top 20 Molecules Generated by MolFinder (ωQ = 0.1).

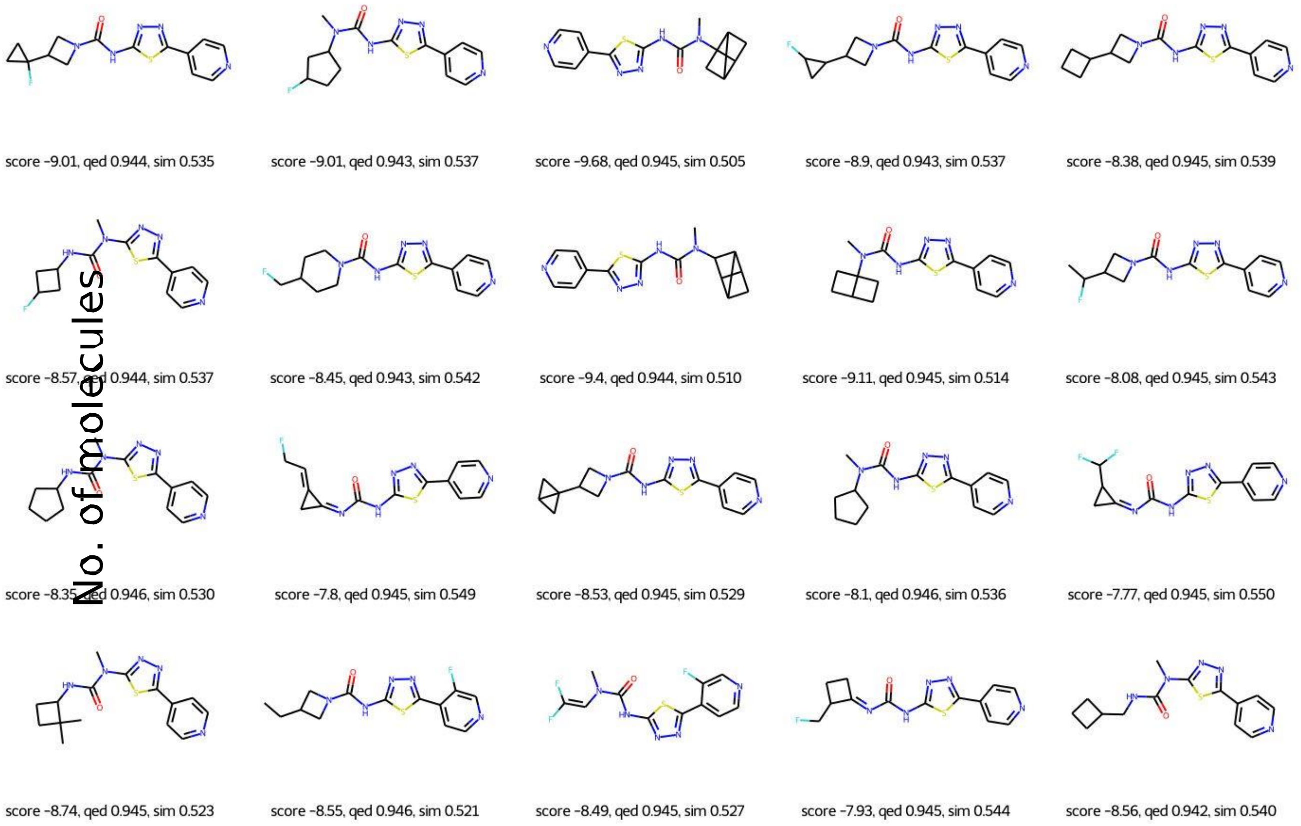

Figure S9. Top 20 Molecules Generated by MolFinder (ωQ = 5.0).

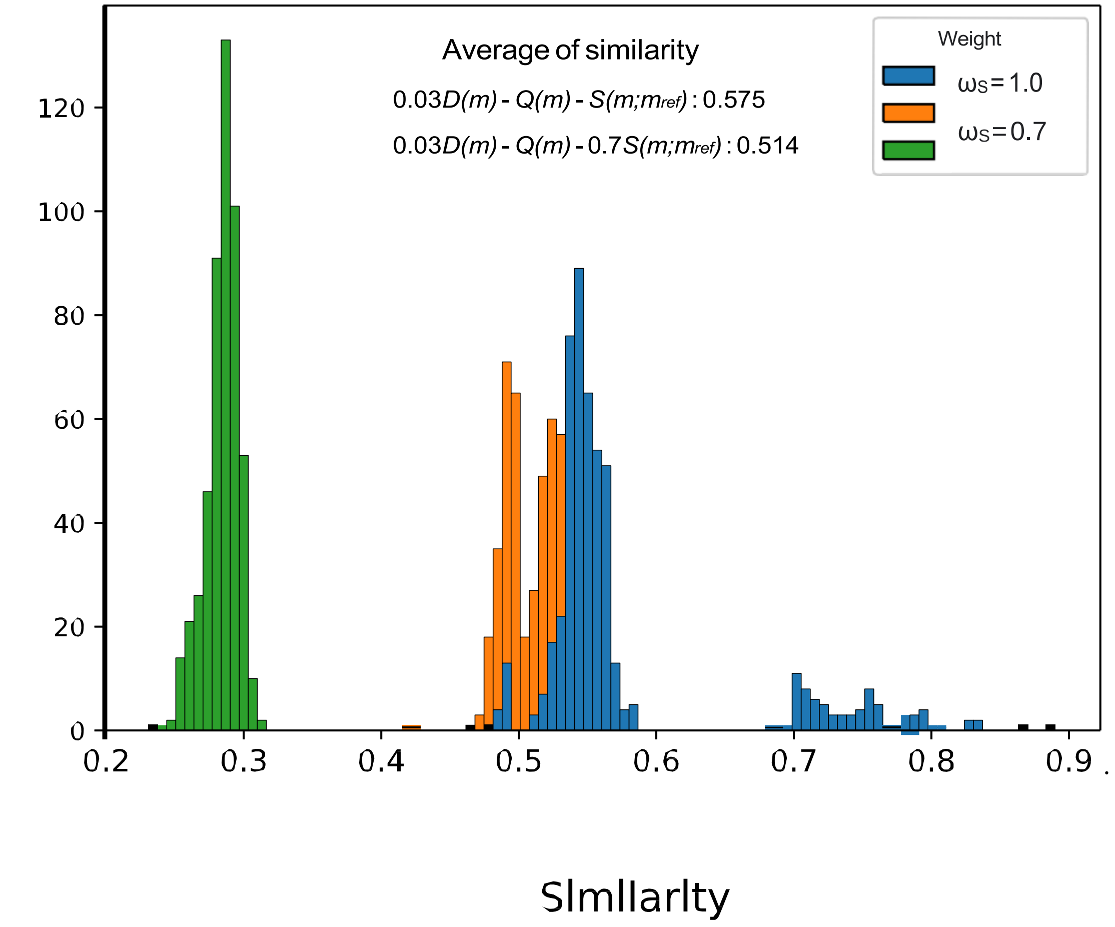

Figure S10. Generated molecules with the known molecules in SureChEMBL while varying the weight coefficient of the similarity term (ωs = 1.0, ωs = 0.7, ωs = 0.4).

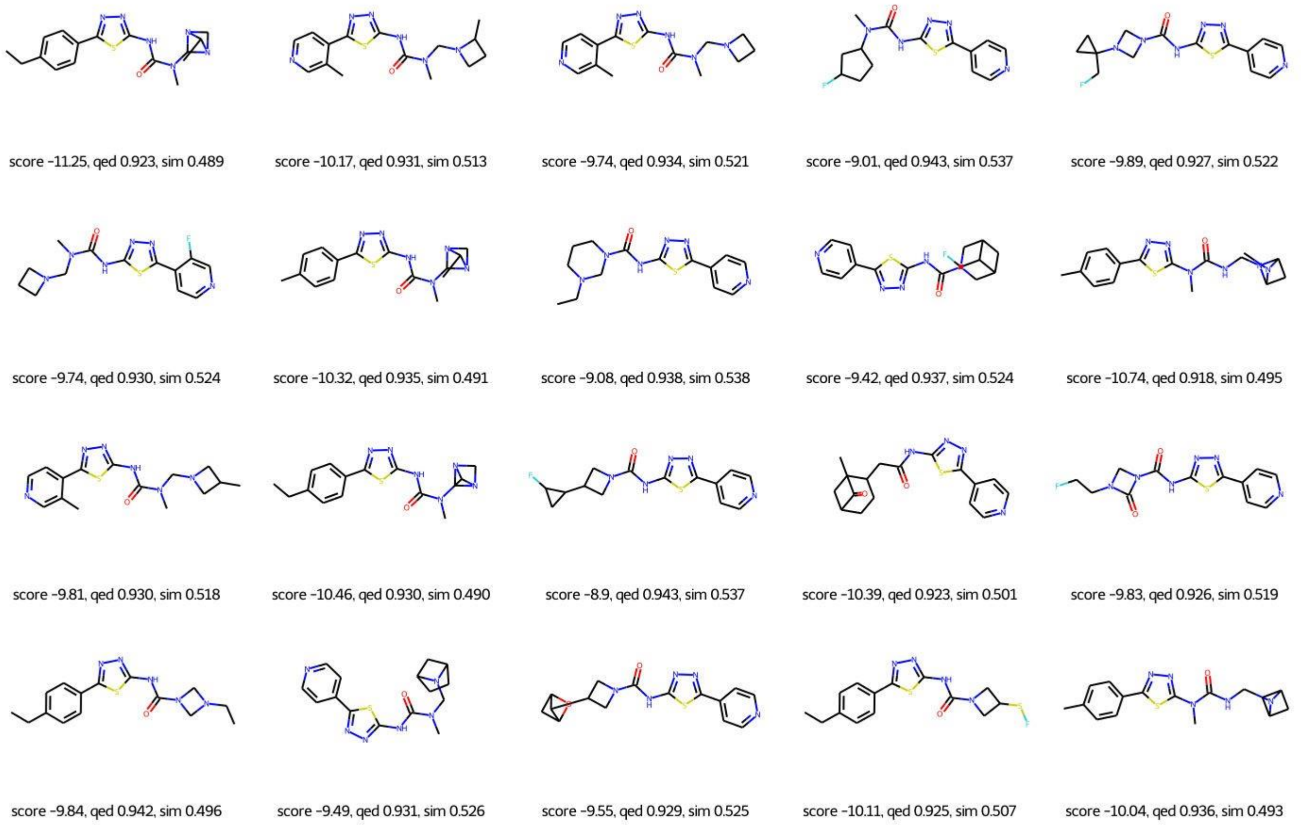

Figure S11. Top 20 Molecules Generated by MolFinder (ωS = 0.7).

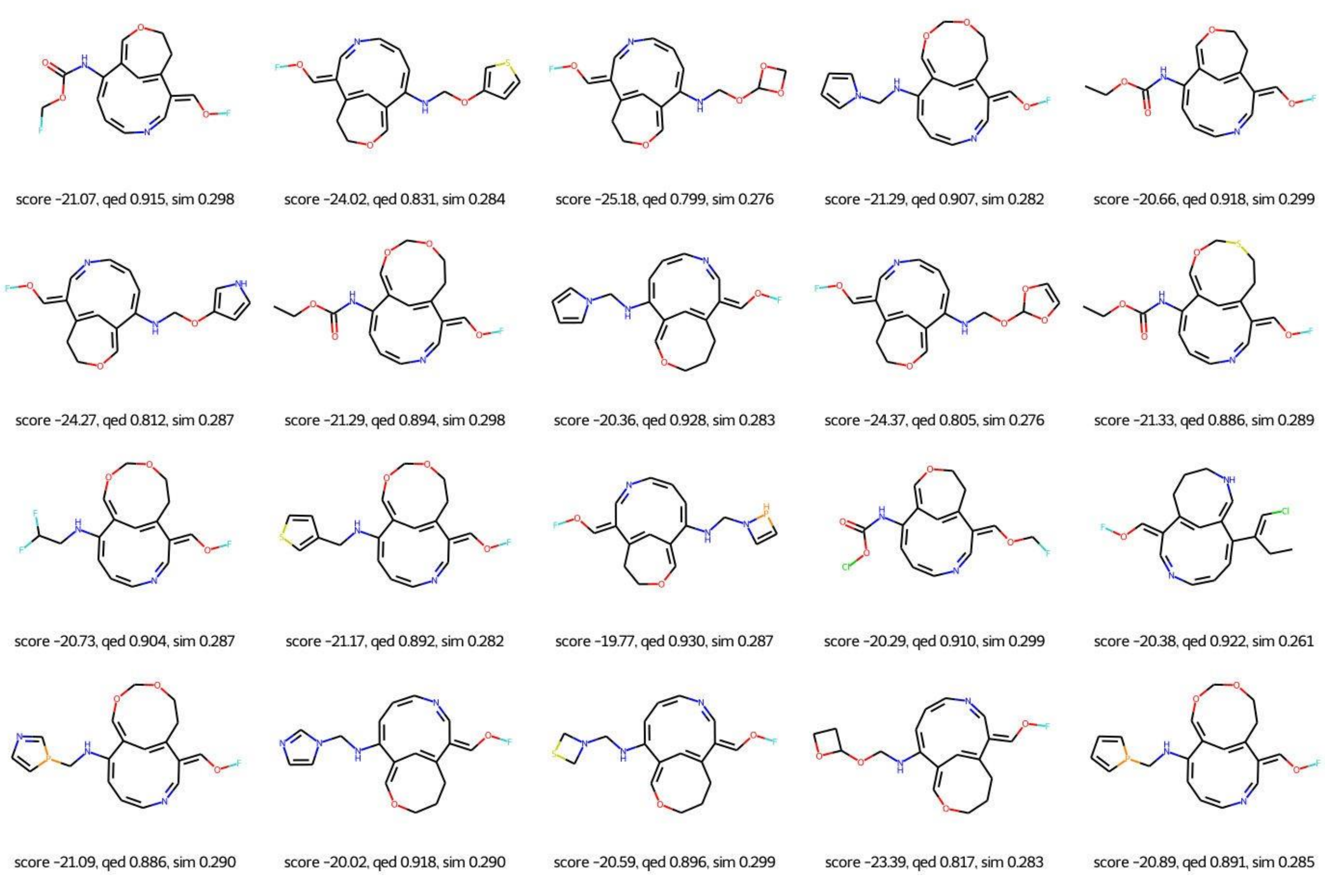

Figure S12. Top 20 Molecules Generated by MolFinder ($\omega S = 0.4$).

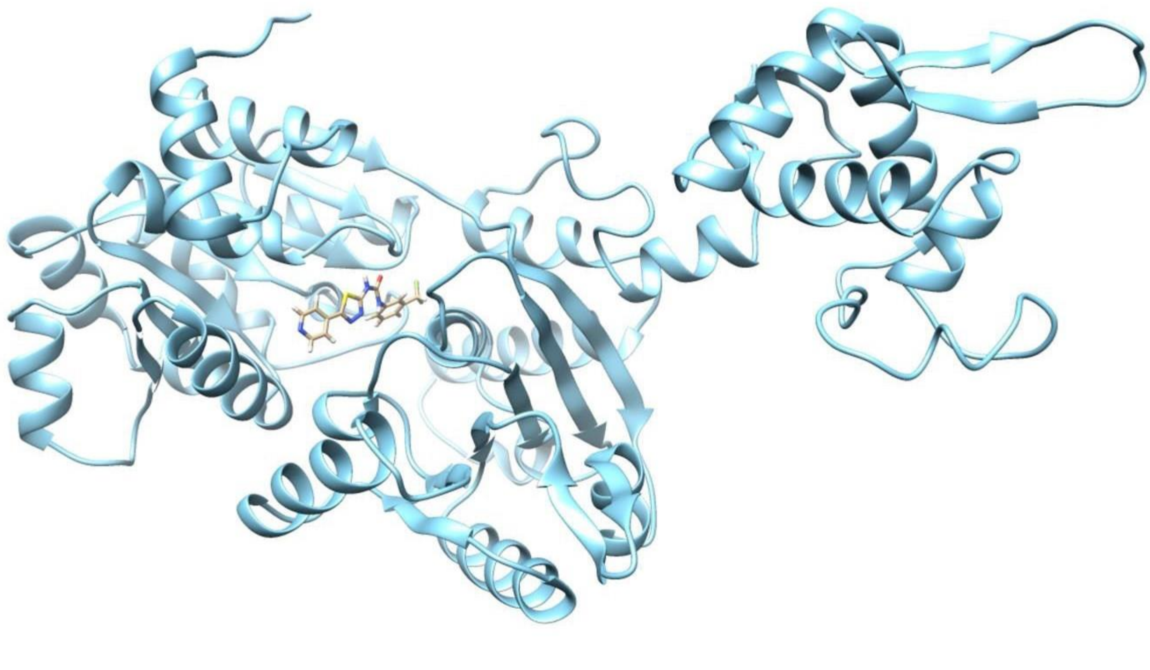

Figure S13. Docking pose of the top first molecule generated using MolFinder.

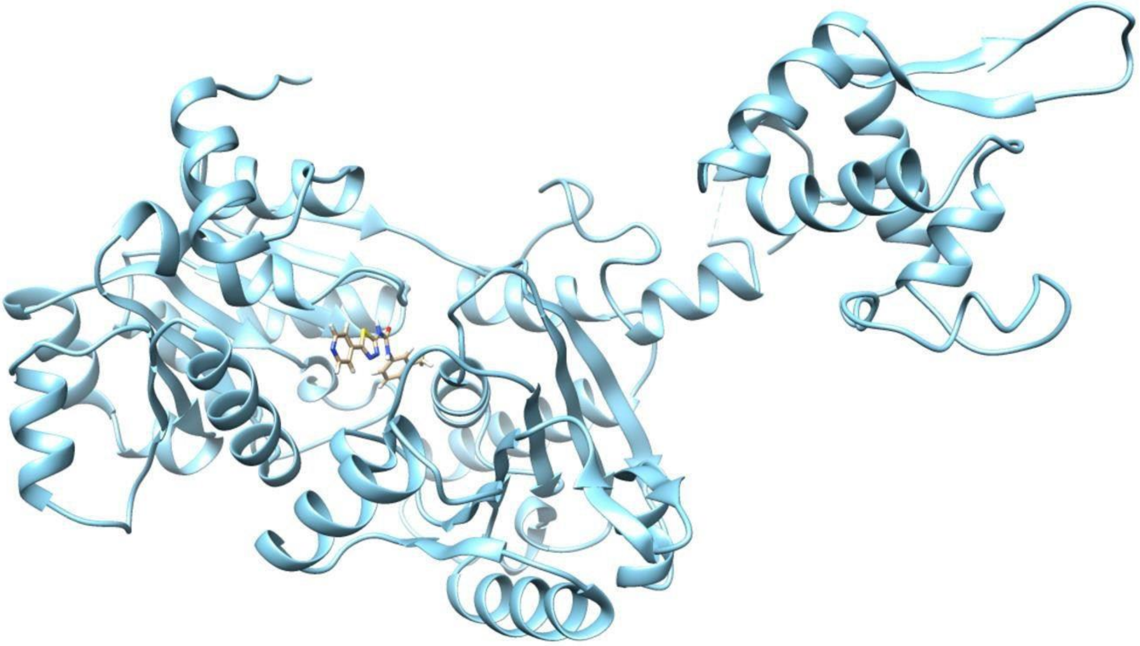

Figure S14. Docking pose of the top second molecule generated using MolFinder.

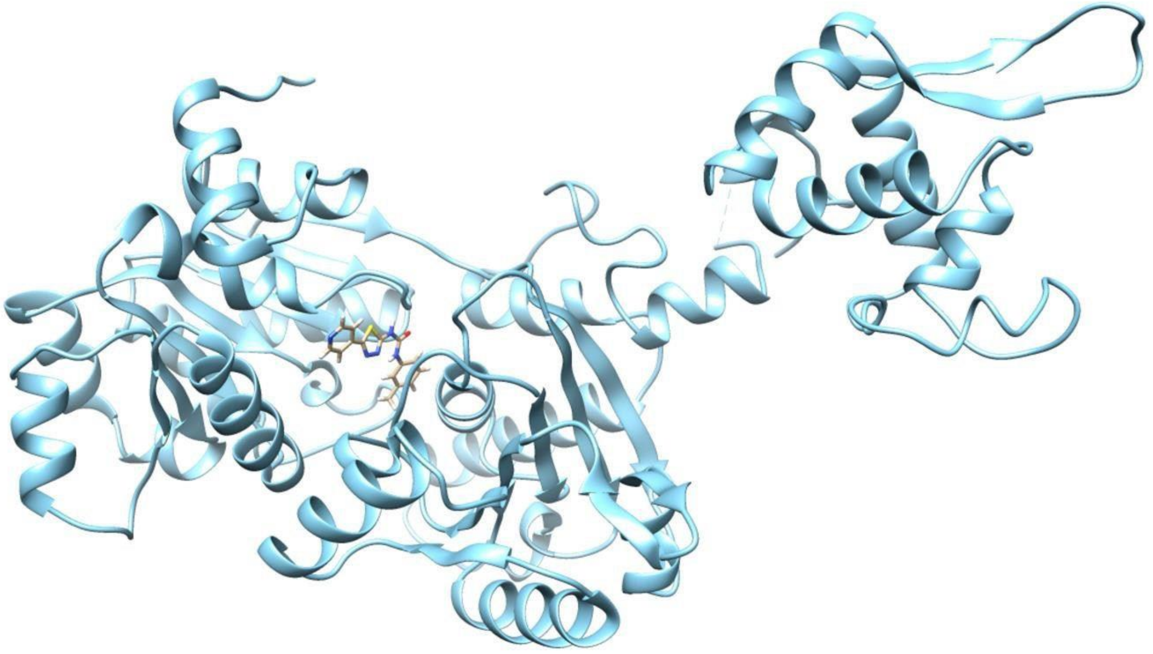

Figure S14. Docking pose of the top third molecule generated using MolFinder.

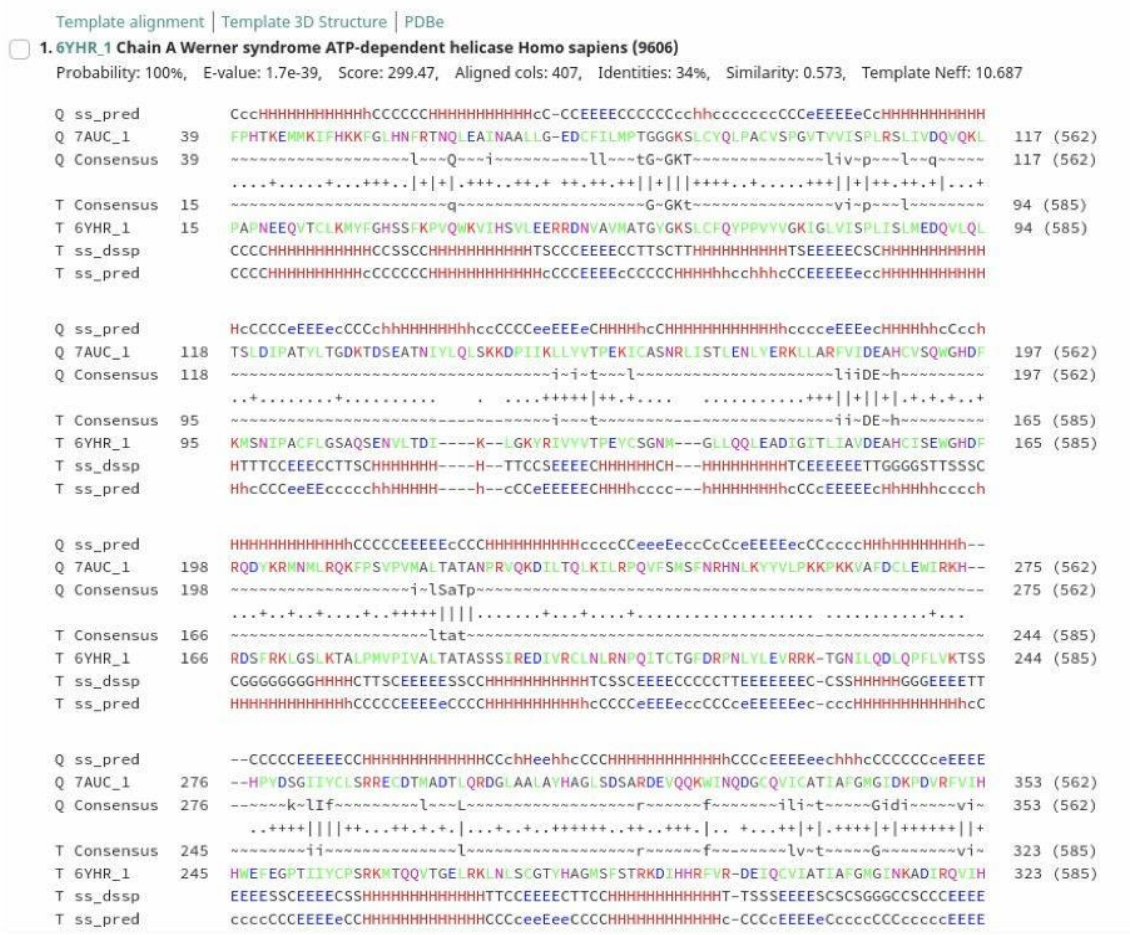

Figure S16. Comparison of ordinal alignment between BLM and WRN.
